# A focus on aromaticity: fuzzier than ever before?

**DOI:** 10.1039/d3sc90075d

**Published:** 2023-05-23

**Authors:** Henrik Ottosson

**Affiliations:** a Department of Chemistry - Ångström, Uppsala University Box 523 Uppsala 751 20 Sweden henrik.ottosson@kemi.uu.se

## Abstract

The field of aromaticity has grown five-fold in the last two decades as revealed by Merino *et al.* in their Perspective “Aromaticity: Quo Vadis” where they ask where the field is heading (*Chem. Sci.*, 2023, https://doi.org/10.1039/D2SC04998H). Numerous computational tools for aromaticity analysis have been introduced and novel classes of molecules that exhibit aromatic (or antiaromatic) features have been explored experimentally. Hence, the aromaticity concept is broader and possibly fuzzier than ever. Yet, earlier it also triggered vigorous debates after periods when new analysis tools emerged, and it survived. Today's debate reveals that the field is vital and that new knowledge is produced. Yet, as much as we ask where the field is moving, we should ask “Aromaticity: Cui Bono?”; who utilizes the aromaticity concept and who benefits from it? Especially, who benefits from it being overly fuzzy and who does the opposite? It is an exciting debate. We should get out of it with a better understanding of the chemical-bonding phenomenon labelled aromaticity.

## Abstract 2

The field of aromaticity has grown extensively in recent decades, and new or unusual forms of aromaticity have been explored. But what exactly is aromaticity? It has always been a fuzzy and disputable concept, yet, is it now fuzzier than ever before?

## Graphical abstract

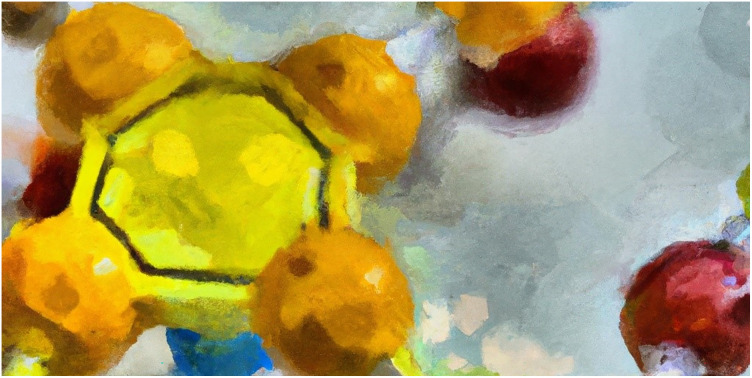

Aromaticity is a concept used within chemistry since the 19th century, even though its meaning and the methods used to explore this phenomenon have changed over time. The history of the aromatic archetype compound, benzene, goes back to its discovery by Michael Faraday in 1825.^1^ In 1865, August Kekulé described his well-known daydream of a snake biting its tail leading him to postulate that benzene has a hexagonal structure.^2^ Some six decades later, in 1929, Kathleen Lonsdale revealed through X-ray crystallography that hexamethylbenzene is indeed a hexagon,^3^ and in 1931 Erich Hückel presented the foundations of his molecular orbital (MO) theory of π-conjugated hydrocarbons providing the basis for the 4*n* + 2 rule of aromaticity.^4^

However, Hückel's MO theory laid dormant until the 1950s when it was discovered *en masse* by the chemistry community. This becomes clear by a comparison of two books from, respectively, 1949 and 1961, in which the properties of aromatic compounds are discussed extensively. Dewar's “Electronic Theory of Organic Chemistry” contains essentially no mention of Hückel's MO theory,^5^ while it has a very prominent position in Streitwieser's “Molecular Orbital Theory for Organic Chemists”.^6^ On the experimental side, NMR spectroscopy, which gave Bloch and Purcell the Nobel prize in Physics in 1952, was introduced and rapidly utilized to explore magnetically induced ring currents in aromatic molecules. In 1965, Breslow launched the counter-concept, antiaromaticity, to represent the opposite of aromaticity and to classify cyclic π-conjugated molecules such as cyclobutadiene and oxirene with 4*n* π-electrons and exceptionally high reactivity.^7,8^

Yet, the aromaticity and antiaromaticity concepts are controversial and the scientific debate has swayed back and forth for decades. In 1970, Heilbronner stated after the opening paper at the Jerusalem Symposium on Aromaticity, Pseudoaromaticity and Antiaromaticity that they had all gathered “*in a symposium on a non-existing subject*”.^9^ And the debate continued. For example, the topic of through-space aromaticity (homoaromaticity) was controversial up until the 1990s,^10–12^ and in recent years, the discussion has partly centered on which methods and criteria should be used for aromaticity assessments (there are geometric, energetic, magnetic and electronic criteria). Clearly, aromaticity and antiaromaticity are concepts that generate very many questions, and consequently, disputes.

As revealed in a Perspective article on this issue, the aromaticity research area, based on the number of published papers, has grown five-fold in the last two decades,^13^ possibly resembling the growth of the field in the 1950s and 1960s. In the Perspective, Gabriel Merino, together with twelve highly established colleagues in the (anti)aromaticity research area, pinpoints a number of issues that need to be dealt with in order to bring more clarity into the field, and they ask where it is moving. A pressing issue is the IUPAC Gold Book description of the aromaticity concept, which is from 1999.

So why do aromaticity and antiaromaticity trigger so much debate? Chemistry has a number of fuzzy concepts,^14^*i.e.*, concepts that cannot be defined precisely but where the corresponding properties (attributes) are still gradable in some way. Several such concepts exist within the domain of chemical bonding, two being aromaticity and antiaromaticity, while other examples are electronegativity, atomic charges and even the chemical bond itself.^14^ These are all concepts that have no associated quantum-mechanical operator. However, fuzzy concepts are not unique to chemistry, and become more common with the complexity of the object. Going to an extreme, the complex societal concept of a city has no unique definition in urban science.^15^

The vast majority of aromatic compounds are regular Hückel-aromatics, but as described by Merino *et al.*, numerous new forms of aromaticity have seen the light-of-day during the last few decades,^13,16^ challenging the traditional description. When asked to illustrate the concept, an AI image generator does not always come up with regular Hückel-aromatic cycles (Fig. 1). Two issues should be of prime concern. First, the constant discovery of new forms of aromaticity leads to the question of what exactly is aromaticity? Are these “new forms of aromaticity” always new or can they be related to already established forms? Or do the molecular features reported represent something other than aromaticity? Instead of discovering “new forms of aromaticity”, it is likely desirable to try to seek unifying patterns that enable a generalization. A second issue of concern is if computational studies carried out in the field of aromaticity are performed with sufficient rigor? This leads to another critical issue: the potential overuse, and sometimes incorrect use, of certain computational tools for aromaticity assessments. Merino and co-authors pinpoint these and similar issues.

**Fig. 1:**
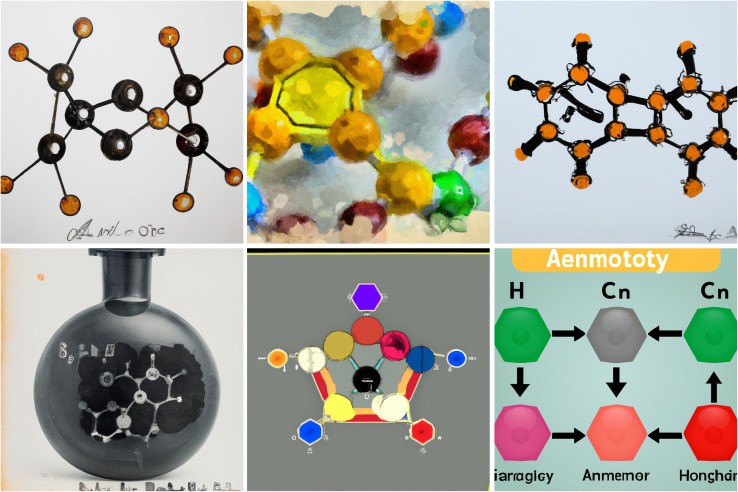
Drawings generated by Shutterstock's AI image generator on the tasks/themes “make a drawing of an aromatic molecule” (all three images in the top row), “molecular aromaticity: fuzzier than ever before?” (bottom row, left), “show aromaticity in some molecules” (bottom row, middle), and “show the chemical concept of aromaticity in four different molecules” (bottom row, right). Reproduced with the permission of Shutterstock.

Yet, the revision of the IUPAC Gold Book definition/description is delicate. Aromatic molecules are found more or less everywhere, from the lignin biopolymers in tree trunks to the heme unit in hemoglobin, the H_3_^+^ ion in the upper atmospheres of Jupiter and Saturn,^17^ and the many pharmaceuticals and their transformation products in our water systems.^18^ Hence, the concept has an exceptionally large number of stakeholders, including the high-school chemistry teacher, the synthetic organic chemist in Big Pharma and the theoretical chemist in academia computing exotic molecules. At the same time, the general IUPAC description of aromaticity should not be at odds with the new and broadly accepted knowledge acquired during the last few decades.

Accordingly, an additional question to the one posed by the Merino and co-authors is: *Aromaticity: Cui Bono*? Who benefits from today's (potentially outdated) IUPAC description, and who does the opposite? Who are the stakeholders of the aromaticity concept today, and who may be so in the future? How to identify the stakeholders, and what are the power relationships between them? Should the revised description be worked out exclusively by experts, or in another way? Can one reach consensus on a new aromaticity description, or are the stakes so high that this will not be possible? Can the present dispute be settled in what may be labelled as a regular public dialogue, or will this just lead to the largest cohort and/or the strongest voices getting their views through?

Today, similar to the previous large shift in the 1950s/60s where both new theory (Hückel MO theory and related models) as well as a new experimental technique (NMR spectroscopy) made entries into the field of aromaticity, we see the introduction of numerous new molecules to which the traditional understanding is not applicable. Furthermore, a number of new quantum chemical methods for (anti)aromaticity assessments provide us with numbers, but if not used properly, they may not provide us with insights but rather the opposite. Thus, the concept might be fuzzier than ever, and it is certainly valid to ask *Aromaticity: Quo Vadis*?

The Perspective article by Merino and co-authors is an excellent starting point for the journey the concepts of aromaticity and antiaromaticity will take us on in the next decade(s). At this point one may ask, is (anti)aromaticity really just one phenomenon or two (or several) related phenomena? There is much that needs to be discovered. With future improved knowledge, we should also be able to utilize these concepts for a number of applications more efficiently, and we will be better able to understand a range of molecular processes. It will be an exciting journey!

## Data availability

Data sharing is not applicable to this article as no datasets were generated or analysed.

## Author contributions

H. Ottosson wrote this article.

## Conflicts of interest

There are no conflicts of interest.

## Supplementary Material
